# Acute Effects of Skeletal Muscle Electrical Stimulation on Central and Lower Extremity Hemodynamics

**DOI:** 10.7759/cureus.62988

**Published:** 2024-06-23

**Authors:** Hajime Tamiya, Hina Kawashiri, Toshiaki Miyamoto, Atsuhiro Tsubaki

**Affiliations:** 1 Department of Exercise Physiology, Institute for Human Movement and Medical Sciences, Niigata University of Health and Welfare, Niigata, JPN; 2 Department of Physical Therapy, Niigata University of Health and Welfare, Niigata, JPN; 3 Department of Physical Therapy, Faculty of Rehabilitation, Kansai Medical University, Osaka, JPN

**Keywords:** shallow femoral artery, hemodynamics, stroke volume, shear rate, cardiac output, neuromuscular electrical stimulation, skeletal muscle electrical stimulation, belt electrode

## Abstract

Introduction: Belt electrode-skeletal muscle electrical stimulation (B-SES) is a treatment prescribed for individuals with difficulty performing exercise therapy that improves muscle strength, exercise tolerance, and glucose metabolism. However, the effects of B-SES on the hemodynamics of the central and lower extremity conduit arteries have not been studied. Therefore, this study compared the acute effects of B-SES on the central and lower extremity conduit arteries in healthy young males.

Methods: This randomized crossover study included nine healthy young males (mean age: 21.0±1.1 years). Participants were assigned to the following experimental conditions, with a washout period of one week: condition 1 included 20 min of electrical stimulation of the lower extremity at the participant’s sensation threshold intensity (Sham, n=9) and condition 2 included 20 min of electrical stimulation of the lower extremity at the maximum intensity the participant can tolerate (B-SES, n=9). The heart rate (HR), stroke volume (SV), cardiac output (CO), mean arterial pressure (MAP), and total peripheral vascular resistance (TPR) were measured as central hemodynamics. The hemodynamics of the lower extremity conduit arteries were measured and calculated for the shallow femoral artery (SFA), including vessel diameter, mean blood flow velocity (MBFV), shear rate (SR), and mean blood flow (MBF) rate. These indices were measured before stimulation (Pre), 10 min after the start of stimulation (Stimulating), and immediately after the end of stimulation (Post). These indices were compared using a repeated two-way analysis of variance.

Results: In B-SES, HR (Pre: 63.2±8.6; Stimulating: 73.7±6.9; Post: 70.0±4.2 bpm, p<0.01), CO (Pre: 5.1±1.0; Stimulating: 6.5±1.5, p<0.01; Post: 6.3±1.2 L/min, p=0.02), and MAP (Pre: 104.0±11.5; Stimulating: 116.4±10.8, p<0.01; Post: 109.6±9.7 mmHg, p=0.02) increased significantly. In addition, B-SES significantly increased MBFV (Pre: 19.2±4.0; Stimulating: 50.5±14.9; Post: 30.1±4.0 cm/s*,* p<0.01), SR (Pre: 118.9±28.8; Stimulating: 302.7±91. 2, p<0.01; Post: 182.1±70.1/s, p=0.02), and MBF (Pre: 382.0±61.5; Stimulating: 1009.6±321.4; Post: 626.8±176.6 mL/min, p<0.01). However, there were no significant changes in SV and TPR.

Conclusions: The findings of this study indicate that B-SES in healthy young males increases CO without increasing SV or TPR and improves the MBFV and SR in the SFA.

## Introduction

Cardiovascular disease (CVD) is the largest contributor to mortality worldwide, accounting for approximately half of the annual deaths attributed to noncommunicable diseases [1]. Notably, considerable scientific literature indicates that increasing physical activity is essential for reducing both the risk of developing and dying from CVD [2-4]. However, many patients are unable to exercise adequately due to increased age-related multimorbidity (comorbidity of multiple chronic conditions) and exercise intolerance [5,6]. Neuromuscular electrical stimulation (NMES) has been used to solve such problems. It is characterized by nonselective muscle contraction and contracts many muscle groups [7], making it effective in preventing skeletal muscle atrophy even when effortful voluntary exercise is difficult [8]. Thus, NMES is suitable for patients who are unable to exercise [9] and can be used as an alternative for patients who cannot participate in exercise-based treatment programs [10].

Recently, belt electrode-skeletal muscle electrical stimulation (B-SES) has attracted particular attention among different types of NMES. B-SES uses belt-type electrodes for electrical stimulation, making it possible to generate muscle contraction not only in a single muscle but also in the entire lower limb [11-13]. In addition, it can stimulate most muscles, including deep areas, without specifying the stimulation site, resulting in significantly less discomfort [14]. These advantages have enabled B-SES to be widely used in a multitude of clinical situations, including that of postoperative orthopedic patients [15], patients undergoing hemodialysis [16-18], patients after allogeneic hematopoietic stem cell transplantation [12], patients with diabetes after lower limb amputation [19], patients in intensive care units [20,21], and patients with severe COVID-19 [22]. However, studies on B-SES have primarily focused on improving muscle mass, strength, physical function, exercise tolerance, and glucose metabolism. To our knowledge, there are no reports on central or lower extremity conduit artery hemodynamics. Conventional NMES of a single muscle improves lower-extremity blood flow and vascular function [23,24]. Therefore, it can be inferred that a B-SES with a wide stimulation range also positively affects hemodynamics. Understanding the physiological mechanisms by which B-SES affects hemodynamics in the central and lower extremity conduit arteries may further expand the indications for B-SES and provide new treatment options to reduce the risk of developing CVD.

To address this gap in the literature, this study aimed to determine the acute effects of B-SES on central and lower extremity conduit artery hemodynamics. We hypothesized that (1) B-SES increases cardiac output (CO) by promoting an increase in venous return due to lower extremity muscle pumping and (2) the increase in CO is accompanied by increased blood flow velocity and shear rate in the lower extremity conduit arteries. Thus, this study will help identify physiological factors that affect vascular function and apply B-SES as a new treatment to reduce the risk of CVD in the future.

## Materials and methods

Study design

**Figure 1:**
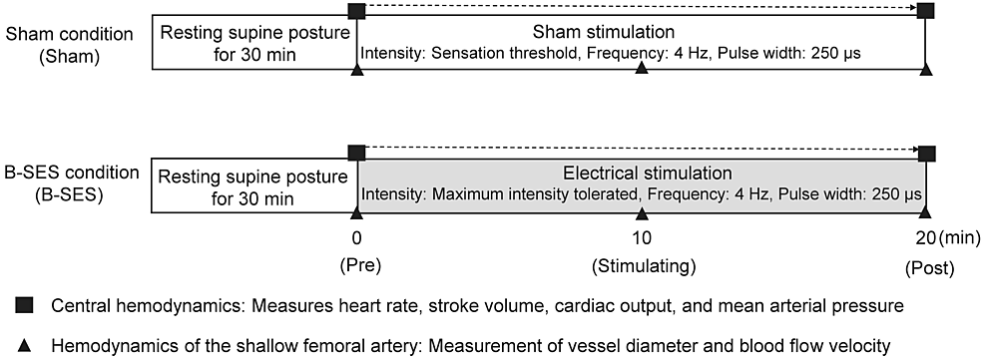
Experimental conditions and measurement index. Two experimental conditions were used in this study. The sham condition (Sham) was stimulated at the intensity of the sensation threshold. In contrast, the B-SES condition (B-SES) was stimulated at the maximum intensity the participant could tolerate. In both conditions, the stimulus frequency was 4 Hz, the pulse width was 250 μs, and the stimulus duration was 20 min. Central hemodynamic measurements were performed throughout the experiment. The hemodynamics of the shallow femoral artery were measured before stimulation, 10 min after the stimulation, and at the end of stimulation. B-SES: belt electrode-skeletal muscle electrical stimulation

This was a randomized controlled crossover trial. Figure 1 shows the experimental conditions and measurement indices used in this study. Two experimental conditions were used in this study. Condition 1: participants received 20 min of electrical stimulation of the lower limb at the sensation threshold intensity (Sham, n=9). Condition 2: 20 min of electrical stimulation of the lower extremity at the maximum intensity the participant could tolerate (B-SES, n=9). Participants were randomly assigned to experimental conditions to eliminate the influence of the order of the experiments. A one-week washout period was allowed to exclude any hemodynamic effects of previous experimental conditions [25].

Study participants

The inclusion criteria for this study were male participants aged ≥18 years and <30 years, with a BMI of ≥18.5 and <25.0. Exclusion criteria were a history of smoking, cardiovascular or metabolic diseases, medications or supplements that may affect vascular function, and a regular exercise routine. Regular exercise habits were defined as moderate physical activity of >150 min weekly or high-intensity physical activity of >75 min weekly [26]. This criterion was used to exclude the influence of daily physical activity on vascular function. Participants self-reported their medication use and medical history. The International Physical Activity Questionnaire (IPAQ-short form) was used to assess physical activity [27]. Nine healthy young males (mean age: 21.0±1.1 years, height: 1.73±0.05 m, weight: 62.8±3.9 kg) voluntarily participated in the study. Table 1 shows the participants’ characteristics.

**Table 1:** Participant demographics. Data are presented as mean±SD. BMI: body mass index; SBP: systolic blood pressure; DBP: diastolic blood pressure; MAP: mean arterial pressure; MVPA: moderate-to-vigorous physical activity (3-6 METs); VPA: vigorous physical activity (>6 METs)

Variable	Value
N	9
Age (years)	21.0±1.1
Height (m)	1.73±0.05
Weight (kg)	62.8±3.9
BMI (kg/m^2^)	21.0±1.0
SBP (mmHg)	121.7±7.2
DBP (mmHg)	72.2±5.9
MAP (mmHg)	88.7±5.9
HR (bpm)	73.0±5.3
MVPA (min/week)	38.3±45.7
VPA (min/week)	0

This study was conducted following the principles of the Declaration of Helsinki and was approved by the Ethics Committee of Niigata University of Health and Welfare (approval number: 19197-231208). The study was registered with the University Hospital Medical Information Network Center (trial ID: UMIN000052787). All participants provided written informed consent before enrollment in this study.

Experimental procedures

The participants were instructed to eat a light meal or not have breakfast for at least 2 h prior to the start of the experiment. In addition, they were instructed not to consume caffeine or alcohol and not to engage in strenuous exercise for at least 24 h before the start of the experiment. All experiments were conducted at room temperature (24-26°C). The participants arrived at the laboratory between 8:00 AM and 10:00 AM. Each measurement was performed after 30 min of supine rest.

NMES Procedure

A belt-electrode electrical stimulator was used for NMES (ZB-SES; Tokyo, Japan: Homer Ion Co. Ltd.). Two belt electrodes were attached to the lumbar region (belt width: 5.5×39.0 cm), bilateral distal thighs (5.5×53.5 cm), and ankles (5.5×30.0 cm). Anode was placed on the distal thigh, cathode on the lumbar region and ankle, and the muscles from the gluteal region to the lower leg were stimulated. Electrical stimulation was performed with an exponentially increasing wave of 250 μs pulses at 4 Hz for 20 min, and all muscle groups were contracted simultaneously. Furthermore, 4 Hz was selected as the stimulation frequency because previous studies have shown that combining 4 Hz B-SES and upper extremity crank exercise positively impacts vascular function [28]. The duty cycle included continuous repeated twitching. The stimulus intensity was defined as the stimulus intensity for each condition. The participants’ pain intensity was rated on a scale of 0-10 using the numerical rating scale (NRS) (10=maximum pain; 0=no pain at all). The NRS target value was set to 7-8. The upper limit of the NRS value was set to 8, and the stimulation intensity was immediately lowered if the NRS value exceeded this limit [8]. In addition, the NRS value changed during electrical stimulation. Therefore, the NRS value was assessed once every 5 minutes, and the electrical stimulation intensity was adjusted each time accordingly.

Central Hemodynamics

The heart rate (HR), stroke volume (SV), and CO were measured using a noninvasive impedance cardiac output meter (PhysioFlow Q-Link, Paris, France: Manatec Biomedical) for each heartbeat and the results were averaged per second [29]. The accuracy of the noninvasive impedance cardiac output meter in normal participants has been previously compared directly with the Fick method [30]. The electrode-applied area was wiped thoroughly with cotton alcohol to optimize the impedance signal. Electrodes were fixed to the skin using a piece of tape to prevent movement during the experiment.

A finger photoplethysmograph (Finapres NOVA; Amsterdam, The Netherlands: Finapres Medical Systems) was used to record the systolic blood pressure (SBP), diastolic blood pressure (DBP), and mean arterial pressure (MAP) beat-by-beat from the middle finger of the left hand using the volume clamp method. Analog data were converted into digital data at a sampling frequency of 1000 Hz using an analog-to-digital converter (PowerLab; Bella Vista, Australia: ADInstruments). Total peripheral resistance (TPR) was calculated from the CO and MAP obtained from the measurements using the below formula [31].

TPR = $\frac{MAP}{CO}$

Central hemodynamics were measured continuously from before to immediately after the end of electrical stimulation. One-minute averages at points before the start of electrical stimulation, 10 min after the stimulation, and immediately after the end were used for the analysis.

Hemodynamics of Conduit Arteries of the Lower Extremities

The vessel diameter and mean blood flow velocity in the shallow femoral artery (SFA) were measured using a general-purpose ultrasound imaging system (ACUSON Juniper; Tokyo, Japan: SIEMENS Healthineers). Furthermore, a 13.3 MHz linear array probe (12L3, ACUSON Juniper; Tokyo, Japan: SIEMENS Healthineers) was placed on the skin 10 cm below the inguinal line [25]. The SFA was selected because it is an easily accessible artery [25]. The angle of incidence of the ultrasound beam was automatically adjusted to 60° and measurements were made using the pulsed Doppler method. The artery was imaged along the short axis, and the arterial diameter was measured during the diastole of the arterial pulse. Images were captured along the long axis, and the average blood flow velocity was measured over three consecutive cycles. The shear rate (SR) and mean blood flow (MBF) in the SFA were calculated from the vessel diameter and mean blood flow velocity (MBFV) using the below equations [32,33].

Shear rate = 4 × mean blood flow velocity/diameter

Mean blood flow = 3.14 × (diameter/2)^2^ × mean blood flow velocity × 60

SFA hemodynamics were measured before commencing electrical stimulation, 10 min after the commencement, and immediately after the end of stimulation.

Statistical analysis

A repeated-measures two-way ANOVA was performed on the two factors of time and between conditions. Tukey's honestly significant difference (HSD) test was used as a post-hoc test when a significant interaction was observed. GraphPad Prism 9.4.1 software (San Diego, CA: GraphPad Software) was used for statistical analysis, and statistical significance was set at 5%. All data are presented as mean±standard deviation.

Sample sizes were calculated using the G power version 3.1.9.7 software (Düsseldorf, Germany: Düsseldorf University). A pilot study was conducted with four healthy young males. The Sham and B-SES conditions were similar to that in the present study, and the effect size was estimated from the change in shear stress before and after stimulation. The effect size was set as 0.62, α: 0.05, 1-β: 0.95, and the sample size was to include nine participants.

## Results

Stimulus current values for each condition were Sham (thigh: 0.9, lower: 0.9 mA) and B-SES (thigh: 2.5±0.6, lower: 2.5±0.5 mA). The participants’ NRS was Sham: 1 (median, range: 0-1) and B-SES: 7 (range: 6-8).

Central hemodynamics

**Figure 2:**
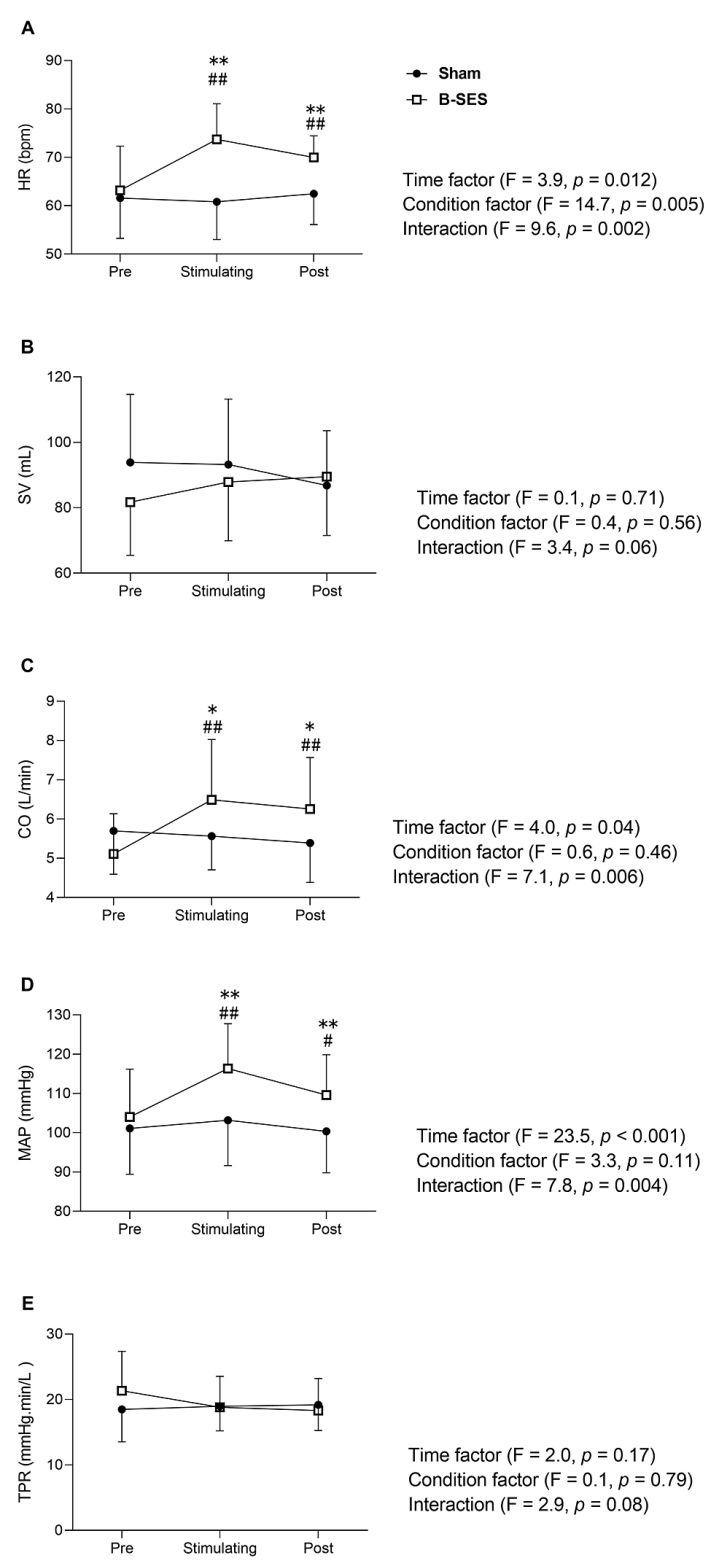
Central hemodynamic changes under Sham (Sham, n=9) and belt electrode-skeletal muscle electrical stimulation (B-SES, n=9) conditions. The images show (A–E) changes over time in HR, SV, CO, MAP, and TPR. Error bars represent SD. #A significant difference from baseline at p<0.05. *A significant difference between conditions at p<0.05. ##A significant difference from baseline at p<0.01. **A significant difference between conditions at p<0.01. HR: heart rate; SV: stroke volume; CO: cardiac output; MAP: mean arterial pressure; TPR: total peripheral vascular resistance; B-SES: Belt electrode-skeletal muscle electrical stimulation

The central hemodynamic changes are shown in Figures 2A-2E. Two-way ANOVA results showed a significant interaction for HR (Figure 2A, p=0.002). B-SES showed significant increases during and after stimulation compared with pre-stimulation (Pre: 63.2±8.6; Stimulating: 73.7±6.9; Post: 70.0±4.2 bpm, p<0.01, respectively). In addition, HR during and after stimulation was significantly higher compared with Sham (Stimulating B-SES: 73.7±6.9 vs. Sham: 60.8±7.4 bpm, p<0.01; Post B-SES: 70.0±4.2 vs. Sham: 62.5±6.0 bpm, p<0.01). However, the sham condition showed no significant change from pre- to post-stimulation (Pre: 61.6±7.8; Stimulating: 60.8±7.4; Post: 62.5±6.0 bpm). Conversely, there was no significant interaction between SV and any other significant changes (Figure 2B).

There was a significant interaction for CO (Figure 2C; p=0.006). In B-SES, there was a significant increase in CO during and after stimulation compared with pre-stimulation (Pre: 5.1±1.0; Stimulating: 6.5±1.5, p<0.01; Post: 6.3±1.2 L/min, p=0.02). Furthermore, CO during and after stimulation was significantly higher compared with Sham (Stimulating B-SES: 6.5±1.5 vs. Sham: 5.6±0.8 L/min, p=0.03; Post B-SES: 6.3±1.5 vs. Sham: 5.4±1.0 L/min, p=0.04). However, the sham condition showed no significant change from pre- to post-stimulation (Pre: 5.7±1.0; Stimulating: 5.6±0.8; Post: 5.4±1.0 L/min).

Similarly, there was a significant interaction for the MAP (Figure 2D; p=0.004). In B-SES, there was a significant increase during and after stimulation compared with pre-stimulation (Pre: 104.0±11.5; Stimulating: 116.4±10.8, p<0.01; Post: 109.6±9.7 mmHg, p=0.02). Furthermore, MAP during and after stimulation was significantly higher compared with Sham (Stimulating B-SES: 116.4±10.8 vs. Sham: 103.2±10.8 mmHg, p<0.01; Post B-SES: 109.6±9.7 vs. Sham: 100.4±10.0 mmHg, p<0.01). However, the sham condition showed no significant change between pre- and post-stimulation (Pre: 101.1±11.0; Stimulating: 103.2±10.8; Post: 100.4±10.0 mmHg). No significant interaction or change was observed in the TPR (Figure 2E).

Hemodynamics of SFA

**Figure 3:**
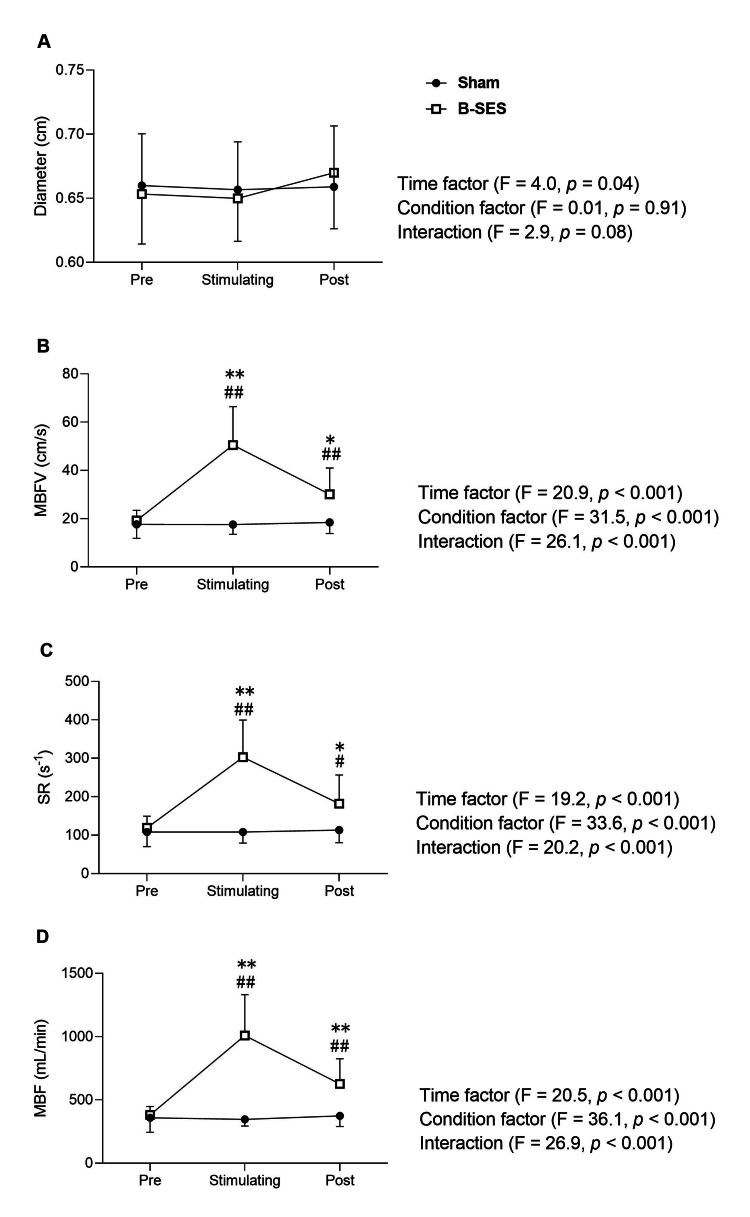
Hemodynamic changes in the shallow femoral artery under sham stimulation (Sham, n=9) and belt electrode-skeletal muscle electrical stimulation (B-SES, n=9) conditions. The images A-D show changes over time in diameter, mean blood flow velocity, shear rate, and mean blood flow, respectively. Error bars represent SD. #indicates a significant difference from baseline at p<0.05. *A significant difference between conditions at p<0.05. ##A significant difference from baseline at p<0.01. **A significant difference between conditions at p<0.01. MBFV: mean blood flow velocity; SR: shear rate; MBF: mean blood flow; B-SES: Belt electrode-skeletal muscle electrical stimulation

Hemodynamic changes in the SFA are shown in Figures 3A-3D. Two-way ANOVA results showed no significant interaction and change in vessel diameter (Figure 3A).

However, there was a significant interaction effect on the MBFV (Figure 3B; p<0.001). B-SES showed a significant increase in MBFV during and after stimulation compared with pre-stimulation (Pre: 19.2±4.0; Stimulating: 50.5±14.9; Post: 30.1±4.0 cm/s, p<0.01). In addition, MBFV during and after stimulation was significantly higher compared with Sham (Stimulating B-SES: 50.5±14.9 vs. Sham: 17.6±3.8 cm/s, p<0.01; Post B-SES: 30.1±10.3 vs. Sham: 18.5±4.4 cm/s, p=0.015). However, the sham condition showed no significant change from stimulus to post-stimulation (Pre: 17.7±5.5; Stimulating: 17.6±3.8; Post: 18.5±4.4 cm/s).

Similarly, there was a significant interaction in SR (Figure 3C; p<0.001). In B-SES, there was a significant increase during and after stimulation compared with pre-stimulation (Pre: 118.9±28.8; Stimulating: 302.7±91.2, p<0.01; Post: 182.1±70.1 s-1, p=0.02). In addition, the SR during and after stimulation was significantly higher compared with Sham (Stimulating B-SES: 302.7±91.2 vs. Sham: 108.0±26.9 s-1, p<0.01; Post B-SES: 182.1±70.1 vs. Sham: 113.3±31.1 s-1, p=0.013). However, the sham condition showed no significant change from pre- to post-stimulation (Pre: 108.7±35.7; Stimulating: 108.0±26.9; Post: 113.3±31.1 s-1).

There was also a significant interaction for the MBF (Figure 3D; p<0.001). B-SES showed a significant increase during and after stimulation compared with pre-stimulation (Pre: 382.0±61.5; Stimulating: 1009.6±321.4; Post: 626.8±176.6 mL/min, p<0.01). In addition, MBF during and after stimulation was significantly higher compared with Sham (Stimulating B-SES: 1009.6±321.4 vs. Sham: 347.3±52.1 mL/min, p<0.01; Post B-SES: 626.8±176.6 vs. Sham: 374.1±81.2 mL/min, p<0.01). However, the sham condition showed no significant change from pre- to post-stimulation (Pre: 358.8±105.0; Stimulating: 347.3±52.1; Post: 374.1±81.2 mL/min).

## Discussion

This study examined the acute effects of B-SES on the hemodynamics of the central and lower extremity conduit arteries. The results showed that in healthy young males, B-SES caused increases in HR, CO, and MAP. It also increased MBFV, SR, and MBF in the SFA. In contrast, B-SES did not affect the SV and TPR, nor did it change the vessel diameter of the SFA. To our knowledge, this is the first study to investigate the acute effects of B-SES on central and lower extremity conduit artery hemodynamics and report their relationship.

Effects on central circulatory dynamics

Contrary to our hypothesis, B-SES did not increase single-beat volume. A previous study predicted that NMES-induced muscle contraction promotes muscle pumping and blood circulation [34], thus increasing the central blood volume during B-SES [28]. The Frank-Starling law states that increased venous return due to enhanced muscle pumping increases SV. However, there was no increase in SV. This may be due to the effects of the stimulation frequency and duty cycles. In previous animal studies comparing venous blood flow with 4-Hz and 50-Hz NMES, we found that the increase in venous blood flow was greater at 50 Hz [35,36]. This is due to the difference in duty cycles between the stimulation protocols [35]. In the present study, the stimulation frequency was 4 Hz, and the cycles consisted of continuous repeated twitching. Therefore, it may be possible to increase SV by increasing the stimulation frequency and providing muscle contraction and relaxation cycles. However, because the frequency and duty cycle were not changed in this study, we cannot make further comments.

Conversely, the HR, CO, and MAP were significantly increased during B-SES. The increment in HR and CO could be due to the higher metabolic load in the B-SES protocol. A study reporting an association between B-SES and oxygen uptake showed that performing B-SES at the maximum intensity tolerated by normal participants resulted in oxygen uptake of approximately 14 mL/min/kg [8]. Since this study was also performed using a similar intensity, HR and CO likely increased due to increased oxygen uptake. Another critical finding is that B-SES in this study significantly increased MAP without changing TPR. The MAP is defined as the product of CO and TPR [31], indicating that the increase in MAP in this study was strongly influenced by increased CO. This may be associated with the vasoconstrictive responses caused by pain and discomfort. Pain and discomfort caused by NMES activate sympathetic nerve activity and induce vasoconstrictive response [37]. However, B-SES is more comfortable because the electrodes are belt-type, stimulating most muscles, including deep areas, without specifying the stimulation site [14]. This may be why the TPR did not increase during electrical stimulation.

The TPR is often used to represent cardiac afterload [38], and excessive afterload increases in patients with high CVD risks can induce cardiac and vascular stress. Therefore, B-SES, which does not increase TPR, may be a less stressful electrical stimulation therapy for vessels in patients with a risk of CVD. However, because the participants in this study were healthy young men, we could not determine whether these results could be extrapolated to patients with a risk of CVD.

Effects of SFA on hemodynamics

B-SES significantly increased the MBFV, SR, and MBF in the SFA. This can be attributed to the increased CO. Exercise causes local accumulation of various metabolites in the active muscle. This then contributes to increased blood flow to the active muscles by inducing redistribution of blood flow through a sympathetic activity [39]. B-SES significantly increases noradrenaline and blood lactate concentrations [8], which strongly supports the occurrence of the muscle metabolic receptor reflex described earlier. The SR and MBF were calculated using the MBFV and vessel diameter. B-SES did not affect vessel diameter in this study. Therefore, the increase in SR and MBF in this study was mainly caused by increased MBFV. SR is the frictional or drag force acting on the vascular lumen, and it has been suggested that it triggers a series of responses, leading to increased vascular endothelial nitric oxide synthase activity [40]. Nitric oxide is crucial in vasodilation and vascular protection under various pathological conditions [41,42]. Therefore, B-SES suggests that it is an effective treatment for reducing the risk of CVD. In addition, the benefit may be particularly high in patients with peripheral arterial disease, considering the increased SR on the SFA.

Furthermore, it has recently been reported that B-SES improves exercise tolerance in patients undergoing hemodialysis who are at risk of CVD [17]. Moreover, combining B-SES and upper limb crank exercise improves vascular endothelial function in healthy participants [28]. Based on these findings, it is likely that B-SES positively affects skeletal muscle and vascular function. Therefore, this study’s increase in shear stress helps explain the physiological mechanisms involved in these results (positive effects on vascular function and patients with vascular disease).

Limitations

This study has some limitations. First, it was limited to healthy young men, making it difficult to extrapolate the results to other demographic groups. Second, B-SES only allows one stimulation pattern; therefore, the effects of frequency and duty cycles cannot be addressed. Future studies should expand the range of participants and examine the effects of multiple stimulus frequencies and duty cycles to better understand the hemodynamic impact of B-SES.

Despite these limitations, this study revealed the effects of B-SES on the central and lower extremity conduit artery hemodynamics. The study results provide fundamental insights into the effects of B-SES on human circulation and contribute to efforts to expand the scope of B-SES and reduce CVD.

## Conclusions

This study showed that B-SES of the lower extremities in healthy young men caused increases in HR, CO, and MAP and significantly increased MBFV, SR, and MBF in the SFA. However, B-SES did not affect SV or TPR. These results suggest that B-SES is an effective electrical stimulation therapy that increases CO without affecting SV and TPR and promotes increased SR and MBF in the SFA. However, because the study participants were healthy young men and the stimulation frequency was limited, future studies should examine the effects of sex differences, diseases, and stimulation frequency.
